# Magnetic silica particles functionalized with guanidine derivatives for microwave-assisted transesterification of waste oil

**DOI:** 10.1038/s41598-021-97097-7

**Published:** 2021-09-01

**Authors:** Petre Chipurici, Alexandru Vlaicu, Ioan Călinescu, Mircea Vînătoru, Cristina Busuioc, Adrian Dinescu, Adi Ghebaur, Edina Rusen, Georgeta Voicu, Maria Ignat, Aurel Diacon

**Affiliations:** 1 Faculty of Applied Chemistry and Materials Science, University Politehnica of Bucharest, 1-7 Gh. Polizu Street, 011061 Bucharest, Romania; 2 National Research and Development Institute for Chemistry and Petrochemistry—ICECHIM, 202 Splaiul Independenţei, 060021 Bucharest, Romania; 3 National Institute for Research and Development in Microtechnologies (IMT-Bucharest), 126 A, Erou Iancu Nicolae Street, P.O. Box 38-160, 023573 Bucharest, Romania; 4 Advanced Polymer Materials Group, University Politehnica of Bucharest, 1-7 Gh. Polizu Street, 011061 Bucharest, Romania; 5 Laboratory of Inorganic Polymers, “Petru Poni” Institute of Macromolecular Chemistry, Aleea Grigore Ghica Voda 41A, 700487 Iasi, Romania

**Keywords:** Catalysis, Chemical engineering, Process chemistry, Materials for energy and catalysis

## Abstract

This study aimed to develop a facile synthesis procedure for heterogeneous catalysts based on organic guanidine derivatives superbases chemically grafted on silica-coated Fe_3_O_4_ magnetic nanoparticles. Thus, the three organosilanes that were obtained by reacting the selected carbodiimides (*N*,*N*′-dicyclohexylcarbodiimide (DCC), *N*,*N*′-diisopropylcarbodiimide (DIC), respectively 1-ethyl-3-(3-dimethylaminopropyl) carbodiimide (EDC) with 3-aminopropyltriethoxysilane (APTES) were used in a one-pot synthesis stage for the generation of a catalytic active protective shell through the simultaneous hydrolysis/condensation reaction with tetraethyl orthosilicate (TEOS). The catalysts were characterized by FTIR, TGA, SEM, BET and XRD analysis confirming the successful covalent attachment of the organic derivatives in the silica shell. The second aim was to highlight the capacity of microwaves (MW) to intensify the transesterification process and to evaluate the activity, stability, and reusability characteristics of the catalysts. Thus, in MW-assisted transesterification reactions, all catalysts displayed FAME yields of over 80% even after 5 reactions/activation cycles. Additionally, the influence of FFA content on the catalytic activity was investigated. As a result, in the case of Fe_3_O_4_@SiO_2_-EDG, a higher tolerance towards the FFA content can be noticed with a FAME yield of over 90% (for a 5% (weight) vs oil catalyst content) and 5% weight FFA content.

## Introduction

Biodiesel can represent a suitable renewable alternative for the direct replacement of standard diesel fuels derived from petroleum sources^1,2^. However, the techno-economic and environmental aspects of the production process must be continuously improved to assure the sustainability of biodiesel in the energy and transportation fuels sectors^3–6^. This can be achieved by decreasing the cost of feedstocks using cheaper raw materials^7–9^, through the increase of productivity by waste reduction and process integration strategies^10–13^, and the implementation of process intensification techniques^14–17^.

Transesterification of vegetable oils using sodium or potassium hydroxide in homogenous catalysis is the preferred route for biodiesel production^18^. However, although the fast reaction rates this procedure presents some major drawbacks such as demands good quality vegetable oil (no water and low free-fatty acids content, usually < 0.5 wt%)^19^ to avoid the soap formation. Furthermore, the catalysts are non-recoverable requiring large volumes of water for their elimination, while corrosiveness affects downstream processing, thus limiting the economic efficiency of the process^11^. The use of heterogeneous catalysis offers a suitable alternative for a greener biodiesel synthesis route, facilitating catalyst separation and reutilization, thus, reducing the amount of waste produced^20–22^. Nevertheless, efficient recovery and longer catalyst lifetime of the catalyst remain challenges that need to be addressed. A good strategy to simplify the catalyst separation is to endow it with magnetic properties^23^.

The use of heterogeneous superbases in the transesterification process has the advantage of high reaction rates under mild conditions as well as catalysts recycling due to facile separation from products^24^. Organic superbases such as guanidines^25^ have attracted increasing attention for application as catalysts in organic synthesis routes due to their potential functionality resulting from their extremely strong basicity. They are also promising active species due to the possibility for facile molecular modification, chemical grafting capacity, possible recyclability, and reduced toxicity. Examples of guanidine derivatives employed in the heterogeneous catalyzed transesterification of vegetable oils include both cyclic derivatives [i.e. 1,5,7-triazabicyclo[4.4.0]dec-5-ene (TBD)]^26–31^ or alkylguanidines^32–35^ [such as tetramethylguanidine (TMG)^36–38^].

The aim of this study was the synthesis and characterization of heterogeneous catalysts based on organic guanidine derivatives superbases chemically grafted on silica-coated Fe_3_O_4_ magnetic nanoparticles. The catalyst preparation involved the synthesis of a magnetic core and the generation of a catalytic active protective shell through the simultaneous hydrolysis/condensation reaction between tetraethyl orthosilicate (TEOS) and three guanidine derivatives silane precursors. The silane precursors were obtained by reacting the selected carbodiimides (*N*,*N*′-dicyclohexylcarbodiimide (DCC)^35^, *N*,*N*′-diisopropylcarbodiimide (DIC), respectively 1-ethyl-3-(3-dimethylaminopropyl) carbodiimide (EDC) with 3-aminopropyltriethoxysilane (APTES). The catalytic activity in conventional and MW-assisted conditions, respectively the catalysts reutilization characteristics for biodiesel production were evaluated at a temperature of 80 °C, a reaction time of 180 min and molar ratio oil/MeOH 1/6. Also, the influence of free fatty acid content on the catalytic activity was also evaluated.

## Experimental

### Materials

The tetraethyl orthosilicate (TEOS) (Aldrich), 3-aminopropyltriethoxysilane (APTES) (Aldrich), *N*,*N*′-dicyclohexylcarbodiimide (DCC) (Aldrich), *N*,*N*′-diisopropylcarbodiimide (DIC), 1-ethyl-3-(3-dimethylaminopropyl) carbodiimide (EDC) (Aldrich), cetyltrimethylammonium bromide (CTAB), lauric acid (Acros), sodium hydroxide (Fluka) were used without purification. Toluene (Merck) was used after distillation and drying on 3 Å molecular sieves. Commercial sunflower oil (Carrefour brand) and methanol (Aldrich) were used in the transesterification reactions without further purifications.

### Methods

#### Synthesis of procedure for (1,3-(dicyclohexyl) 2-(3-(triethoxysilyl)propyl) guanidine) (Si-DCG), (1,3-(diisopropyl) 2-(3-(triethoxysilyl) propyl) guanidine) (Si-DIG) and (1- ethyl, 3 (3-(3-dimethylamino propyl)) 2-(3-(triethoxysilyl)propyl) guanidine) (Si-EDG)

**Scheme 1:**
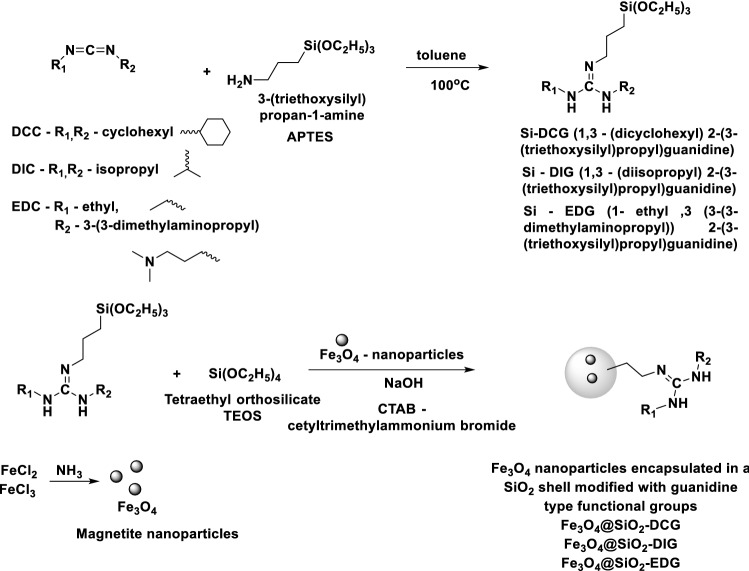
Reactions pathways for the preparation of the catalysts.

The synthesis procedure was based on a previous literature example^35^ and it was expanded for EDC and DIC. It involves the addition of amino group of APTES to the three carbodiimides derivatives (DCC, EDC and DIC) (Scheme 1).

In a 50 mL round bottom flask, 10 mmoles of the starting material carbodiimide (2.1 g *N*,*N*′ diciclohexyl carbodimides (DCC) or 1.3 g *N*,*N*′-diisopropylcarbodiimide (DIC), or 1.55 g 1-ethyl-3-(3-dimethylaminopropyl) carbodiimide (EDC), respectively) were dissolved in 20 mL of dry toluene under a nitrogen blanket. To this solution 10 mmoles (2.21 g) of 3-aminopropyltriethoxysilane (APTES) were added and the mixture was kept under continuous stirring at 100 °C for 24 h. The toluene was removed using a rotary evaporator at reduced pressure and yellowish oils were obtained and used directly for the preparation of the catalysts without any further purifications.

#### Synthesis procedure of the magnetic nanoparticles Fe_3_O_4_

The magnetic Fe_3_O_4_ nanoparticles were prepared by a chemical co-precipitation method^31,39^. 18 mmoles FeCl_3_·6H_2_O (4.864 g) and 8.9 mmoles FeCl_2_ (1.134 g) were dissolved in 100 mL of bidistilled water. The solution was degassed by nitrogen bubbling for 15 min at room temperature under mechanical stirring (350 rpm) while being immersed in an ultrasonic water bath (40 kHz and 400 W). The US power absorbed by the solution was determined calorimetrically as 15.2 W with a corresponding power density of 0.152 W/mL). Then, 10 mL of 25% NH_4_OH were added to this solution and the reaction mixture remained under mechanical stirring and nitrogen atmosphere at room temperature for 1 h. The black precipitate was separated from the solution using magnetic decantation and was subsequently washed thoroughly (10 times) with deionized water and acetone. The resulting black solid was dried under vacuum at room temperature for 5 h, yielding 2.10 g of magnetic nanoparticles.

#### Synthesis procedure for the magnetic catalysts

In a 250 mL two-necked round bottom flask equipped with mechanical stirring, 0.55 g cetyltrimethylammonium bromide (CTAB) were dissolved in 200 mL of water. In the solution obtained 1 g of magnetic nanoparticles were dispersed to serve as core for the catalyst generation. After the preparation of the dispersion, the solution was heated at 90 °C and 1.5 mL of NaOH 2 mol/L were added and stirred for 15 min. Then, 22.4 mmoles (5 mL) TEOS were added while continuously stirring the reaction mixture. After 10 min from the introduction of TEOS (the solution became opalescent) the silane-guanidine precursor (Si-DCG, Si-DIG or Si-EDG respectively) was introduced and the reaction was stirred for an additional 4 h at 90 °C. After, the catalyst was isolated by magnetic assisted decantation, washed thoroughly with water, ethanol, and acetone. The final products were dried at 70 °C for 24 h, affording the magnetic catalysts (Fe_3_O_4_@SiO_2_-DCG, Fe_3_O_4_@SiO_2_-DIG and Fe_3_O_4_@SiO_2_-EDG).

### Transesterification procedure

The MW-assisted transesterification reactions were performed using a Biotage Initiator microwave equipment with a single-mode applicator (https://selekt.biotage.com/hubfs/ORGANIC/Premium%20Content_Documents/Initiator/PPS299.v4%20-%20Microwave%20synthesis%20brochure.pdf?hsLang=en). The reactions were carried out in 20 mL glass reactors capable of resisting at 10 bars. The reactor was charged with methanol (2.26 g), catalyst (0.35 g), and oil (10 g) (commercial sunflower oil) to ensure an oil: methanol molar ratio of 1:6 and a catalyst content of 3.5% (wt% versus oil). The microwave power was 400 W for a few seconds and then 40–60 W to maintain the reaction temperature at 80 °C for 180 min under magnetic stirring (780 rpm). The transesterification reactions for the conventional heating were performed in the same glass reactor using a silicon heating bath, the same temperature, stirring rate and reaction time. For the free fatty acid (FFA) content influence study, lauric acid was used together with the commercial sunflower oil to obtain the desired FFA content.

After the reaction, the catalyst was removed by magnetic separation (using a neodymium magnet) and decanting. The glycerol was separated by settling or centrifugation. The upper layer formed was washed with acidified water and dried over calcium chloride (this wet method is used only at laboratory scale, for industrial process, dry washing may be considered^11^). Then, the organic phase was processed with a rotary evaporator to remove unreacted methanol.

Fatty acid methyl ester content was determined by GC analysis using a HP 6890 gas chromatograph with HP-INNOWAX 19091N-133 column 30 m × 250 μm, according to European Standards^40^. The concentration of FAME was calculated from integration of the chromatograms using the area of the peaks. The esters content was calculated using the Eq. (1).

1 $$C\left(\%\right)= \frac{(\sum Ai-As)\times Cs\times Vs\times 100}{(As\times m)}.$$

Unde: ∑*Ai*—total peak area from the FAME C_14:0_ to C_24:1_; *As*—peak area of internal standard—methyl heptadecanoate; *Cs*—concentration, in mg/ mL, of the internal standard methyl heptadecanoate; *Vs*—volume, in mL, of the internal standard solution, methyl heptadecanoate; *m*—mass, in mg, of the sample.

The transesterification reaction yield was calculated using the Eq. (2).

2 $$yield\left(\%\right)=\frac{mass\,\, of\,\, pure\,\, FAME \,\,obtained\,\,(g)}{theoretical\,\, mass\,\, of\,\, FAME\,\, obtained\,\, from\,\, the\,\, reaction\,\, (g)}\times 100$$

The activation of the catalyst consisted in the dispersion of the magnetic particles before each reaction in an alkaline solution (methanolic solution of NaOH 1 M, for 5 min ultrasound assisted—GT SONIC Professional Ultrasonic Cleaner, VGT-1620 T model, US Power 50 W) followed by magnetic separation and then washing with methanol up to a pH of 8 for the residual methanolic solutions. Before the transesterification reaction or between reactions in the case of reutilization of the catalyst, the catalyst was dried.

### Characterization

FTIR analysis was performed using a Bruker VERTEX 70 spectrometer using 32 scans with a resolution of 4 cm^−1^ in 4000–600 cm^−1^ region. The catalysts and intermediates were analyzed using the KBr pellet technique for the fresh catalyst and attenuated total reflection (ATR) technique for the used catalysts.

The morphology of the catalysts was also evaluated by FEI Nova NanoSEM 630 Scanning Electron Microscope (FEG-SEM) using an ultra-high-resolution detector (TLD detector) at an acceleration voltage of 10 kV and a working distance of 5 mm. The elemental identification and quantification within the catalyst particles were performed with the Element energy dispersive spectroscopy (EDS) system (Smart Insight AMETEK). The Energy-dispersive X-ray spectroscopy spectra were acquired at an acceleration voltage of 10 kV.

The thermogravimetric analyses (TGA) of the catalysts were performed using a Netzsch TG 209 F3 Tarsus equipment considering the next parameters: nitrogen atmosphere flow rate 20 mL min^−1^; samples mass: ∼ 3 mg; temperature range: room temperature − 700 °C; heating rate: 10 °C min^−1^ in an alumina (Al_2_O_3_) crucible.

To measure the basicity of the solid catalysts, samples of 0.05 g were shaken for 15 min in 0.02 M aqueous HCl (5 mL), and the remaining acid was then titrated with an aqueous solution of NaOH in the presence of phenolphthalein^41^. The base strength of the samples (H_) was assessed using different acid–base indicators^42^. The indicators used in the study (their p*K*_a_ values are given in the parentheses): phenolphthalein (8.2), 2,4,6-trinitroaniline (12.2), 2,4-dinitroaniline (15.0), 4-chloro-2-nitroaniline (17.2), 4-nitroaniline (18.4) and 4-chloroaniline (26.5).

The digestion of the samples was performed using the speedwave XPERT microwave digestion system (Berghof Products—Instruments GmbH) involving a digestion protocol based on HCl/HF mixture (see Table 1). The HF excess have been neutralized with H_3_BO_3_. To determine the total nitrogen of the digested mixtures, the organic nitrogen to nitrate digestion by persulfate has been performed. The contents of the total nitrogen (TN) in digested samples were determined by using a Multi N/C 3100 TOC Analyzer (Analytik Jena, Germany) equipped with a CLD chemiluminescence detector for TN analysis. The nitrogen content was calculated as mg of nitrogen per gram of solid sample.

**Table 1:** Digestion protocol.

Sample name	Mass (g)	HCl, 37% (mL)	HF, 48% (mL)	K_2_S_2_O_8_ (mg)	H_3_BO_3_,40 g/L (mL)
Blank sample (digestion solution)	0	6	1	5	12
Fe_3_O_4_-SiO_2_-EDG	0.2271	6	1	5	12
Fe_3_O_4_-SiO_2_-DCG	0.2167	6	1	5	12
Fe_3_O_4_-SiO_2_-DIG	0.2236	6	1	5	12

*The digestion mixture was diluted: 1 mL mixture with 49 mL of ultrapure water.

## Result and discussion

**Scheme 2:**
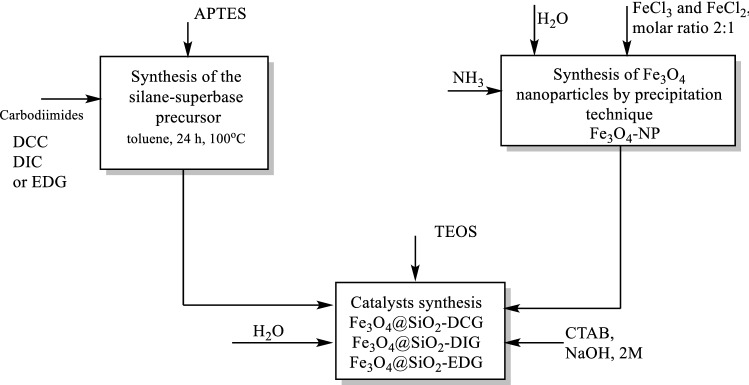
Schematic representation for the synthesis of the magnetic catalysts (Fe_3_O_4_@SiO_2_-DCG, Fe_3_O_4_@SiO_2_-DIG and Fe_3_O_4_@SiO_2_-EDG).

The first step of this study consisted in the synthesis of magnetic SiO_2_ particles with three different guanidine derivatives, organic superbases. The synthesis strategy adopted involved the generation of Fe_3_O_4_ nanoparticles by precipitation technique starting from a mixture of FeCl_3_ and FeCl_2_ using ammonia under ultrasonic treatment. The magnetic nanoparticles were then coated with a silica shell using TEOS and a silane-superbase precursor, obtained by reacting different carbodiimides with APTES. Thus, magnetic particles presenting a SiO_2_-superbase shell were obtained through a one-pot synthesis approach (Schemes 1 and 2).

**Figure 1:**
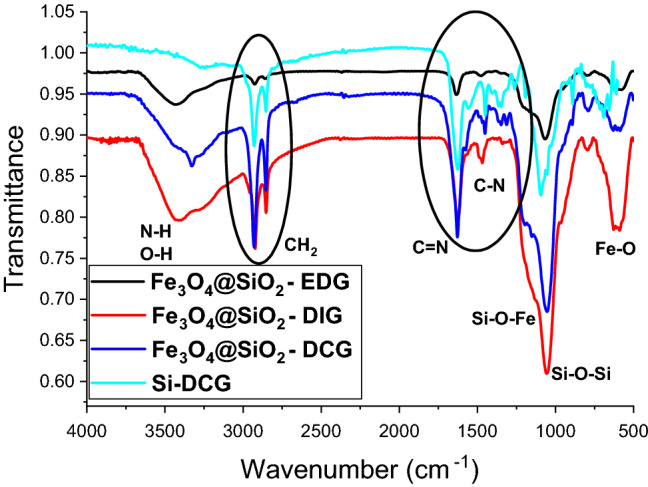
FTIR spectra for the magnetic catalysts (Fe_3_O_4_@SiO_2_-DCG, Fe_3_O_4_@SiO_2_-DIG, and Fe_3_O_4_@SiO_2_-EDG).

The successful synthesis of the magnetic catalyst particles was investigated by FTIR analysis (Fig. 1). The presence of the organic bases is confirmed by the characteristics signals for the organic derivatives such as a large band at 3400–3000 cm^−1^ specific for O–H and N–H stretching^31,43^, 2930 cm^−1^ and 2850 cm^−1^ specific for the asymmetric and symmetric C–H vibration^44^, 1630 cm^−1^, and 1448 cm^−1^ attributed to C=N and C–N (from guanidine) stretching^31,35,38,45^. Further, the presence of the magnetite and silica shell leads to the presence of a specific stretching vibration band of the siloxane groups (Si–O) at 1051 cm^−1^ and an intense band due to Fe–O bond vibrations^46^ split into two peaks at 635 and 588 cm^−1^. This band splitting has been previously associated with the nanometric size of magnetic nanoparticles (Fe_3_O_4_) and was observed for all of the prepared catalysts^31^.

**Figure 2:**
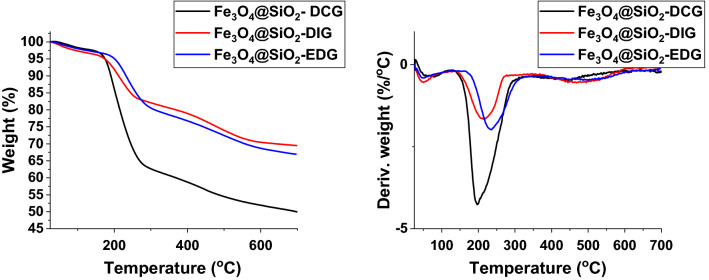
TGA and DTG curves for the magnetic catalysts (Fe_3_O_4_@SiO_2_-DCG, Fe_3_O_4_@SiO_2_-DIG and Fe_3_O_4_@SiO_2_-EDG).

TGA analysis was employed to investigate the thermal stability of the catalysts and to assess the amount of organic superbase covalently bonded to the silica shell (Fig. 2). The initial weight loss observed in all cases is attributed to physiosorbed water and solvent molecules on the surface of the catalysts (less than 2% in all cases). The second weight-loss step is comprised of two consecutive weight-losses, and it can be attributed to the detachment of the organosilane and the guanidine derivatives from the silica shell. The DTG curves indicated that the second weight loss step begins at temperatures above 195 °C and the highest thermal stability was observed for Fe_3_O_4_@SiO_2_-EDG. The high temperature confirms a chemical attachment between the organosilane derivatives and the silica. The weight loss for this step is approximately 36%, 15%, and 18.8% for Fe_3_O_4_@SiO_2_-DCG, Fe_3_O_4_@SiO_2_-DIG, and Fe_3_O_4_@SiO_2_-EDG, respectively. This difference in weight loss is following the difference in the molecular weight of the superbases, indicating that a comparable molar concentration of superbase was successfully attached to the silica.

**Figure 3:**
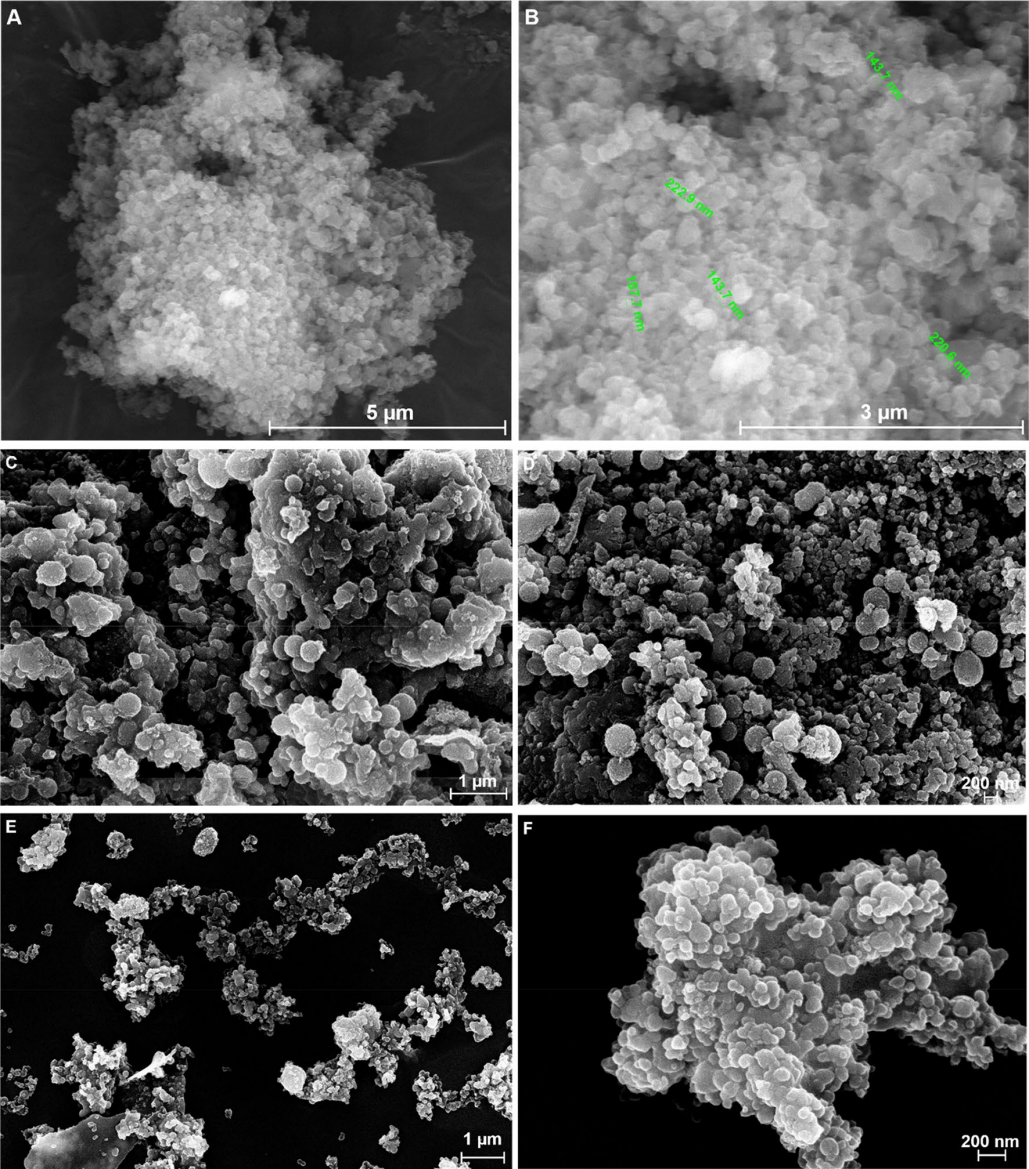
SEM images of the magnetic catalysts: Fe_3_O_4_@SiO_2_-DCG (**A**, **B**), Fe_3_O_4_@SiO_2_-DIG (**C**, **D**) and Fe_3_O_4_@SiO_2_-EDG (**E**, **F**).

The morphology of the catalysts was investigated by SEM analysis to assess the influence of the organosilane derivative on the SiO_2_ shell. Considering that the SiO_2_ shell formation involves the simultaneous hydrolysis/polycondensation of both TEOS and the organosilane derivative, leading to a functionalized silica shell. The SEM images revealed the development of spherical-shaped catalysts particles with particle sizes of 100–200 nm (Fig. 3). The catalyst particles dimensions vary only slightly between the samples with the size decreasing in the order Fe_3_O_4_@SiO_2_-DCG > Fe_3_O_4_@SiO_2_-DIG > Fe_3_O_4_@SiO_2_-EDG, which can be related to an increase of the basicity of the superbases and the presence of a tertiary amine group in the case of Fe_3_O_4_@SiO_2_-EDG. The relative agglomerated characteristics of the catalyst particles can be related to the high content of guanidine derivatives^43^.

**Figure 4:**
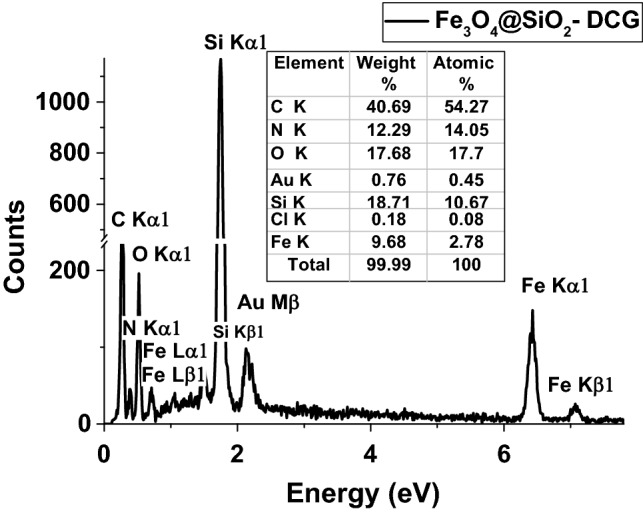
EDS spectra of Fe_3_O_4_@SiO_2_-DCG catalyst.

The magnetic catalyst particles were also investigated by EDS analysis to ascertain their surface chemical composition (Fig. 4). The analysis confirmed the presence of Fe, O, Si, C and N atoms which would be in accordance with a core–shell structuring of the material and confirms the presence of organic derivatives on the surface of the Fe_3_O_4_@SiO_2_-DCG catalyst. The higher C content denoted by the EDS analysis compared to the TGA analysis results suggests that the organic derivatives are predominantly present at the surface of the particles. This could be explained by the hydrophilic characteristic of the organic functional groups that influence the formation and structuring of the micelles during the silane precursors hydrolysis step. Further, the characteristic signals for Fe may indicate a somewhat deficient covering of magnetite particles by the silica shell or a covering by a very thin layer.

To evaluate the nitrogen amount in the bulk of the catalysts, total nitrogen (TN) content analysis was performed. The results are presented in Table 2. Compared with the EDS information the TN analysis indicates the organic derivatives are predominantly present at the surface of the catalysts which will improve their activity.

**Table 2:** Total organic nitrogen determined for the solid catalysts.

Sample name	Nitrogen conc. in the catalyst (wt%)	Nitrogen conc. in the catalyst (mg/g)
Fe_3_O_4_-SiO_2_-EDG	2.72	27.2
Fe_3_O_4_-SiO_2_-DCG	0.38	3.8
Fe_3_O_4_-SiO_2_-DIG	1.23	12.3

A qualitative base strength (H_) assessment of the samples was performed using Hammet indicator method. The value obtained were between 15.0 ≤ H ≤ 17 for all the catalysts^35,42^. However, due to the initial brown shade of the solid catalysts the change of color of the solid catalyst is very difficult to be properly evaluated. The titration of the total base indicated the 0.74 ± 0.02, 0.80 ± 0.04 and 0.88 ± 0.02 mmol/g_cat_ for Fe_3_O_4_@SiO_2_-DCG, Fe_3_O_4_@SiO_2_-EDG and Fe_3_O_4_@SiO_2_-DIG, respectively. The amount of total base varies only slightly between the samples. The results are in accordance with other literature examples^35^ and compared to the TN values indicate the contribution of other basic sites besides the guanidine derivatives.

The BET analysis was performed to assess the textural properties of the magnetic catalysts (Table 3). The analysis revealed comparable specific surfaces with a slight increase in the case of Fe_3_O_4_@SiO_2_-DIG. Also, the pore volume is comparable between Fe_3_O_4_@SiO_2_-DIG and Fe_3_O_4_@SiO_2_-EDG, while an increased size was registered for Fe_3_O_4_@SiO_2_-DIG and Fe_3_O_4_@SiO_2_-DCG. Considering that the reaction takes place in liquid media, the values for the specific surface area are reasonable, while an increased pore size should facilitate the adsorption/desorption and diffusion processes.

**Table 3:** Textural properties of the magnetic catalysts.

Sample	BET surface area, m^2^/g	BJH pore volume, cm^3^/g	BJH pore size, nm
Fe_3_O_4_@SiO_2_-DCG	41.79	0.078	8.13
Fe_3_O_4_@SiO_2_-DIG	58.58	0.082	6.38
Fe_3_O_4_@SiO_2_-EDG	44.14	0.068	6.85

**Figure 5:**
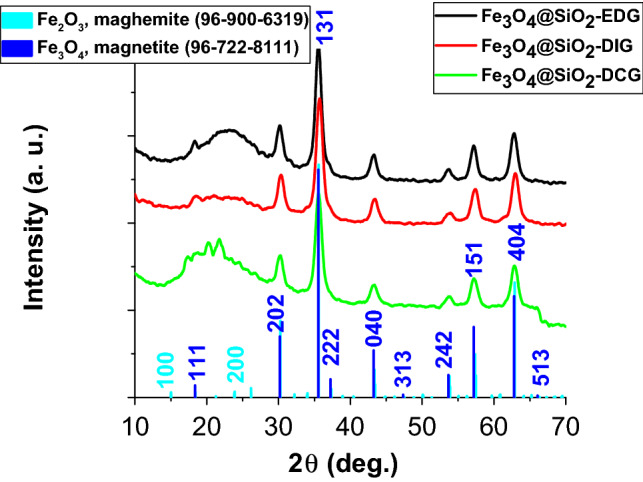
XRD analysis of the magnetic catalysts (Fe_3_O_4_@SiO_2_-DCG, Fe_3_O_4_@SiO_2_-DIG, and Fe_3_O_4_@SiO_2_-EDG).

The XRD analysis of the magnetic catalysts (Fig. 5) indicates the presence of a spinel-structured oxide identified as either magnetite (Fe_3_O_4_) or a mixture between magnetite (Fe_3_O_4_) and maghemite (γ-Fe_2_O_3_) (in the case of Fe_3_O_4_@SiO_2_-DCG). No characteristic peaks could be attributed to the silica shell, possibly due to its small thickness and amorphous characteristics.

Different preparation and use of guanidine derivatives anchored on silica gel as catalysts for biodiesel production were previously reported in the literature^35,37,39^. The yield of biodiesel was within 90–95% at 80 °C and 180 min of reaction time with the use of dicyclohexylguanidine but using a methanol/oil molar ratio in the range of 20–30. In the case of tetramethylguanidine, the yield of biodiesel was 86% at similar reaction conditions.

**Figure 6:**
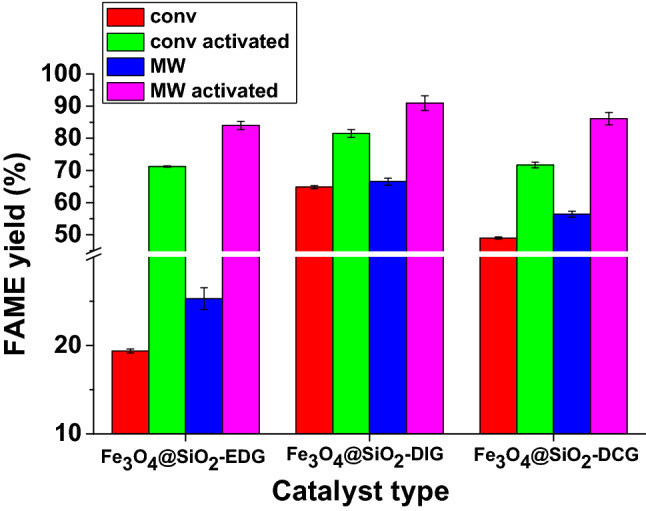
FAME yield depending on the catalyst type and transesterification reaction characteristics (conventional or MW assisted) and on the activation of the catalyst (temperature 80 °C; reaction time 180 min; molar ratio oil/MeOH 1/6; 3.5% weight ratio catalyst vs. oil; 780 rpm).

Our results of the investigation of the catalytic activity of the synthesized magnetic catalysts for the biodiesel synthesis and the possibility for process intensification using microwave (MW) irradiation are presented in Fig. 6. The reactions were performed in our case at a temperature of 80 °C, a reaction time of 180 min, molar ratio oil/MeOH 1/6, a 3.5% weight ratio catalyst vs. oil, and a stirring rate of 780 rpm. The catalytic activity variation was in the order: Fe_3_O_4_@SiO_2_-DIG > Fe_3_O_4_@SiO_2_-DCG > Fe_3_O_4_@SiO_2_-EDG. Additionally, the process intensification capacity of microwaves can be noticed for all catalysts. Ferrite-containing catalysts are materials with very good microwave energy absorption properties^47^. Even if the size of the catalyst particles is very small, a selective heating effect of the catalyst particles is possible, which may justify the favorable effect of the microwaves^48^.

Nevertheless, the catalyst activation stage proves to have a dramatic role in the catalyst activity. The activation of the catalyst aims to assure a deprotonated C=N bond from guanidine derivatives prior to the transesterification process, which promotes the deprotonation of methanol during the first stage of the base-catalyzed transesterification process^2,49^. Thus, by activation, all catalysts display FAME yields of over 70% at a temperature of 80 ºC, a reaction time of 180 min, molar ratio oil/MeOH 1/6, a 3.5% weight ratio catalyst vs. oil (Fig. 6). The process intensification induced by the MW-assisted transesterification was also observed for the activated catalysts. Thus, FAME yields of over 80% were obtained for all catalysts with the yield reaching 91% in the case of Fe_3_O_4_@SiO_2_-DIG. The catalytic activity variation of both MW-assisted transesterification reaction using the activated catalysts followed the same trend Fe_3_O_4_@SiO_2_-DIG > Fe_3_O_4_@SiO_2_-DCG > Fe_3_O_4_@SiO_2_-EDG.

**Figure 7:**
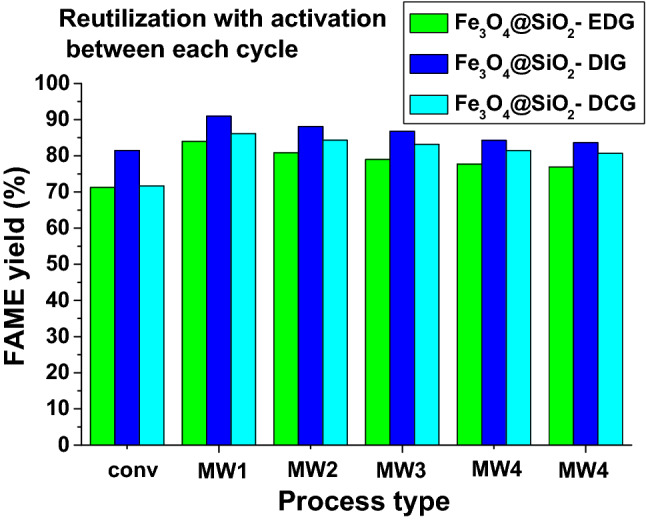
FAME yield for the recycling experiments for the magnetic catalysts with an activation stage between each cycle (activation before each reaction; temperature 80 ºC; reaction time 180 min; molar ratio oil/MeOH 1/6; 3.5% weight ratio catalyst vs. oil; 780 rpm).

Previous examples of guanidines derivatives use as catalysts for vegetable oil transesterification described high reaction yields in homogeneous^50^ or heterogeneous systems^32,35^, but the reuse of heterogeneous catalysts usually provided a challenge due to the leaching^18,30,31,34,51^ from the support and require covalent bonding to the support^37^. In our case, through the simultaneous hydrolysis/polycondensation of both TEOS and of the organosilane, the chemical attachment of the guanidine derivatives to the catalysts structures is assured, therefore the leaching of the superbase problems should be eliminated. To confirm this, catalyst reutilization experiments were performed under MW irradiation for the process intensification (Fig. 7). The results show that during five reaction cycles the FAME yield decreases gradually from 83.96%, 90.95%, and 86.12% to 76.9, 83.6%, and 80.7% for Fe_3_O_4_@SiO_2_-EDG, Fe_3_O_4_@SiO_2_-DIG, and respectively Fe_3_O_4_@SiO_2_-DCG. This decrease in the FAME yield can be explained by a decrease of the active area through adsorption of intermediate species that are not removed during the activation stage or by some leaching due to guanidine derivatives weakly attached to the surface of the catalysts^37^.

**Figure 8:**
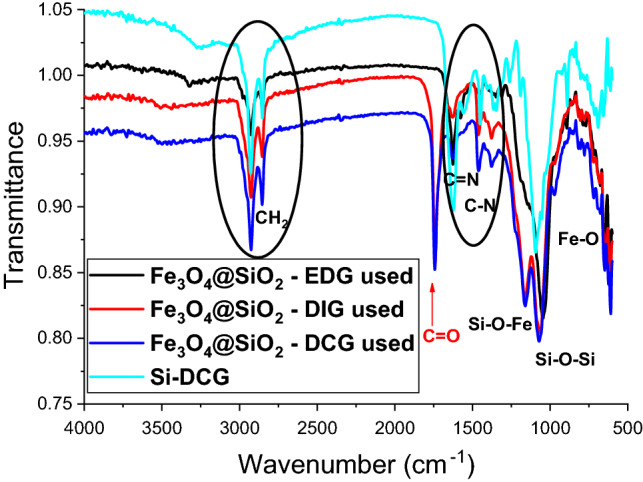
FTIR spectra for the magnetic catalysts (Fe_3_O_4_@SiO_2_-DCG, Fe_3_O_4_@SiO_2_-DIG, and Fe_3_O_4_@SiO_2_-EDG) after the reaction and before the re-activation step.

FTIR analysis was employed to investigate the catalysts after the transesterification reaction (Fig. 8). The analysis of the spectra reveals the presence of a supplementary vibration band with a maximum at 1743 cm^−1^ specific for the vibration of C=O functional group for Fe_3_O_4_@SiO_2_-DCG and Fe_3_O_4_@SiO_2_-DIG catalysts. Interestingly, in the case of Fe_3_O_4_@SiO_2_-EDG the supplementary signal is not present. The signal can be attributed to the adsorption of biodiesel or intermediate mono-, diglyceride or free fatty acids species to the catalysts surface. The other vibrations specific for the guanidine derivatives remained unchanged indicating good stability of the catalysts.

**Figure 9:**
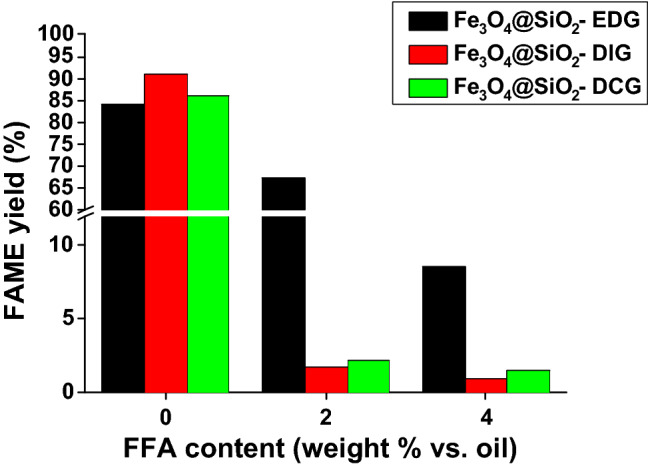
FAME yield depending on the FFA (wt% vs. oil) content and catalyst type (activated catalyst; temperature 80 °C (MW irradiation); reaction time 180 min; molar ratio oil/MeOH 1/6; 3.5% weight ratio catalyst vs. oil; 780 rpm).

Considering possible differences in the absorption characteristics of the catalyst our next objective consisted in the investigation of the catalytic activity in the case of a starting material with a higher content of free fatty acids (FFA). The capability of a catalyst to be employed for the transesterification of oils with a higher FFA content is highly desirable when considering as starting material waste cooking oils. The results presented in Fig. 9 indicate a dramatic drop of the FAME yield with the increase of FFA content in the case of Fe_3_O_4_@SiO_2_-DIG and Fe_3_O_4_@SiO_2_-DCG. However, in the case of Fe_3_O_4_@SiO_2_-EDG, a higher tolerance towards the FFA content can be noticed with a FAME yield of 67.38% (for a 3.5% (weight) vs oil catalyst content) at a 2% weight FFA content. This activity can be correlated with the presence of a tertiary amine in addition to the guanidine functionality in the case of Fe_3_O_4_@SiO_2_-EDG. Thus, the functional groups present in Fe_3_O_4_@SiO_2_-EDG make possible the existence of an intramolecular hydrogen bond and intrinsic proton affinity characteristic in the case of Fe_3_O_4_@SiO_2_-EDG^52^.

**Figure 10:**
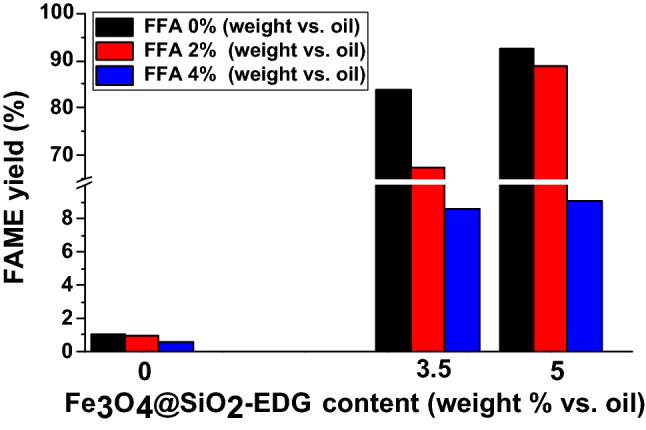
FAME yield depending on the Fe_3_O_4_@SiO_2_ catalyst content (wt% vs oil) and FFA content in the oil (activated catalyst; temperature 80 °C (MW irradiation); reaction time 180 min; molar ratio oil/MeOH 1/6; 780 rpm).

To further investigate the activity of Fe_3_O_4_@SiO_2_-EDG at high FFA content in the starting material, experiments with different concentrations of catalyst were performed (Fig. 10). The results confirm that increasing the catalyst concentration leads to a higher FAME yield. However, at a higher concentration of FFA, the catalyst activity also decreases affording FAME yield below 10%. Thus, although the tertiary amine helps at extending the tolerance of the catalyst the intramolecular hydrogen bond at high FFA content leads to a deactivation of the catalyst active sites. However, this deactivation is reversible and by washing with alkaline methanol solution the catalyst can be reactivated with comparable results.

## Conclusions

In conclusion, this study presented the synthesis, characterization, and catalytic activity evaluation of three heterogeneous catalysts based on guanidine derivatives both in conventional and MW-assisted transesterification reaction for biodiesel production. The catalyst consisted of magnetic particles encapsulated in a silica shell containing the guanidine derivative for their facile recovery from the reaction media. The catalysts were characterized by FTIR, TGA, SEM, BET and XRD analysis confirming the successful covalent attachment of the organic derivatives on the silica shell.

The catalytic activity evaluation aimed to confirm the good activity of the catalysts with FAME yields of over 70% being obtained at an oil/methanol ratio of 1/6, at 80 °C and 180 min reaction time using conventional heating. The activity of the catalysts showed improvement and good stability in MW-assisted transesterification reactions, all catalysts displaying FAME yields of over 80% after 5 reaction/activation cycles. The catalytic activity variation was in the order: Fe_3_O_4_@SiO_2_-DIG > Fe_3_O_4_@SiO_2_-DCG > Fe_3_O_4_@SiO_2_-EDG. Thus, the catalysts displayed good activity, stability, and reusability at a relative low reaction temperature for heterogeneous catalysts and low oil/methanol molar ratio. Furthermore, the influence of FFA content on the catalytic activity was investigated. Thus, in the case of Fe_3_O_4_@SiO_2_-EDG, a higher tolerance towards the FFA content can be noticed with a FAME yield of over 90% (for a 5% (weight) vs oil catalyst content) and 5% weight FFA content. Thus, the combination of a guanidine functional group and tertiary amine in EDG allows for a higher tolerance towards FFA content. Thus, the facile synthesis procedure for the catalysts developed as well as their activity, stability, recyclability, and capability to process less expensive oil feedstocks and permit the improvement of biodiesel techno-economic parameters.
